# Prognostic value of transforming growth factor-beta in patients with colorectal cancer who undergo surgery: a meta-analysis

**DOI:** 10.1186/s12885-017-3215-7

**Published:** 2017-04-04

**Authors:** Xin-lin Chen, Zhuo-qun Chen, Shui-lian Zhu, Tian-wen Liu, Yi Wen, Yi-sheng Su, Xu-jie Xi, Yue Hu, Lei Lian, Feng-bin Liu

**Affiliations:** 1 School of Basic Medical Science, Guangzhou University of Chinese Medicine, Guangzhou, China; 2 The First Clinical College, Guangzhou University of Chinese Medicine, Guangzhou, China; 3 Guangdong Province Hospital of Chinese Medicine, Guangzhou University of Chinese Medicine, Guangzhou, China; 4 Department of Colorectal Surgery, Sun Yat-sen University, Guangzhou, China; 5 The First Affiliated Hospital, Guangzhou University of Chinese Medicine, Guangzhou, China

**Keywords:** Colorectal cancer, Transforming growth factor-beta, TGF-β, Prognosis, Meta-analysis

## Abstract

**Background:**

Transforming growth factor-beta (TGF-β) is associated with a higher incidence of distant metastasis and decreased survival. Whether TGF-β can be used as a prognostic indicator of colorectal cancer (CRC) remains controversial.

**Methods:**

The Medline, EMBASE and Cochrane databases were searched from their inception to March 2016. The studies that focused on TGF-β as a prognostic factor in patients with CRC were included in this analysis. Overall survival (OS) and disease-free survival (DFS) were analysed separately. A meta-analysis was performed, and hazard ratios (HR) with 95% confidence intervals (CI) were calculated.

**Results:**

Twelve studies were included in the analysis, of which 8 were used for OS and 7 for DFS. In all, 1622 patients with CRC undergoing surgery were included. Combined HRs suggested that high expression of TGF-β had a favourable impact on OS (HR = 1.68, 95% CI: 1.10–2.59) and DFS (HR = 1.11, 95% CI: 1.03–1.19) in CRC patients. For OS, the combined HRs of Asian studies and Western studies were 1.50 (95% CI: 0.61–3.68) and 1.80 (95% CI: 1.33–2.45), respectively. For DFS, the combined HRs of Asian studies and Western studies were 1.42 (95% CI: 0.61–3.31) and 1.11 (95% CI: 1.03–1.20), respectively.

**Conclusions:**

This meta-analysis demonstrates that TGF-β can be used as a prognostic biomarker for CRC patients undergoing surgery, especially for CRC patients from Western countries.

**Electronic supplementary material:**

The online version of this article (doi:10.1186/s12885-017-3215-7) contains supplementary material, which is available to authorized users.

## Background

Colorectal cancer (CRC) is one of the most common malignancies worldwide. In terms of frequency, colorectal cancer ranked third in North America and Europe and fifth in Asia among malignant diseases [1, 2]. More than 1.2 million patients are diagnosed with CRC every year, and of these, more than 600,000 die [3]. The 5-year survival rate for patients with metastatic CRC is 10-15%, whereas for patients with non-metastatic CRC, the rate is 40-90% [4]. Recent advances in genetic and molecular characterisation of CRC have yielded a set of prognostic and predictive biomarkers that aid in the identification of patients at a higher risk for disease recurrence and progression [5]. Some investigators have reported that drugs that target signalling pathways involved in tumourigenesis; for example, cetuximab for wild-type K-ras CRC [6], and bevacizumab for CRC [7], improve survival of patients with CRC over chemotherapy alone [6–8].

The transforming growth factor-β (TGF-β) family includes TGF-β1, TGF-β2 and TGF-β3, which are expressed during tumour progression [9]. TGF-β has been shown to be a critical regulator and is considered a tumour suppressor because it inhibits cell cycle progression and stimulates apoptosis in the early stages of cancer progression [10, 11]. However, TGF-β may transform from an inhibitor of tumour cell growth to a stimulator of growth and invasion in advanced stages of CRC [12–14]. TGF-β can modulate cancer-related processes, such as cell invasion, distant metastasis, and modification of the microenvironment in advanced stages of CRC.

Many studies have been performed to assess the prognostic value of TGF-β in patients with CRC, but the conclusions of these studies have been inconsistent. Some studies demonstrated that high expression of TGF-β was associated with worse survival of patients with CRC [15, 16] whereas some studies failed to show any statistically significant association between high expression of TGF-β and survival in patients with CRC [10, 17]. Thus, it is unclear whether high expression of TGF-β is associated with worse survival in CRC patients. To our knowledge, no meta-analysis has been performed to assess the prognostic effects of TGF-β. Therefore, our goal was to combine all the results from published studies, after which we systematically evaluated the essential roles of TGF-β in colorectal cancer.

## Methods

### Search strategy

The meta-analysis was conducted in accordance with the PRISMA statement [18]; the PRISMA 2009 Checklist is shown in additional files (Additional file 1). Two reviewers independently conducted a systematic literature search of the following databases from the database inceptions to March 8th, 2016: PubMed, EMBASE, and the Cochrane Central Register of Controlled Trials. The search included the following terms:

1. “colorectal” OR “large intestine” OR “large bowel” OR “colon” OR “colonic” OR “rectal” OR “rectum”
2. “cancer” OR “carcinoma” OR “tumor” OR “tumour” OR “neoplasm” OR “cancers”
3. “TGF-β” OR “TGF-β1” OR “transforming growth factor”
4. “prognosis” OR “prognoses” OR “prognostic” OR “predictive” OR “biomarker” OR “marker” OR “survival” OR “survive” OR “Cox” OR “Log-rank” OR “Kaplan-Meier”

The search was not limited by language. The specific search strategy is shown in the additional files (Additional file 2).

The systematic reviews and meta-analyses on TGF-β and CRC were manually reviewed for potentially relevant studies. Relevant studies were also retrieved using Google scholar with the following search terms: “colorectal cancer, colon cancer, or rectal cancer”, “TGF-β, TGF-β1 or transforming growth factor” and “prognoses, predictive or survive”.

### Inclusion criteria

The inclusion and exclusion criteria included the following four aspects. (1) Patients diagnosed with CRC (including colon cancer and rectal cancer) were eligible for inclusion. Patients with different clinical stages, histological types, or treatment methods were all included but patients with other diseases were excluded. (2) The expression of TGF-β (protein, mRNA) was measured by polymerase chain reaction (PCR), immunohistochemistry (IHC) or enzyme-linked immunosorbent assay (ELISA) in primary CRC (including colon cancer, or rectal cancer) tissues. (3) The association between TGF-β and patient prognosis (i.e., overall survival [OS], disease-free survival [DFS], and/or relapse-free survival [RFS]) was investigated; the hazard ratio (HR), 95% confidence interval (CI), or the relevant information was provided. (4) A full paper was published in English. The eligible studies included cross-sectional studies, cohort studies, and even randomised controlled trials. When the same author reported multiple studies from the same patient population, the most recent study or the most complete study was included. The studies published in abstract form were considered only if sufficient outcome data could be retrieved from the abstract or from communication with the authors.

### Study selection

Duplicate studies from different databases were identified, and the remaining abstracts were read for eligibility by two independent authors (ZQC and SLZ); the studies with inconsistent results were reviewed by the third author (XLC). The full texts of potentially eligible studies were retrieved and reviewed independently by two authors (ZQC and SLZ). Any disagreements were recorded and resolved by consensus under the guidance of the third author (XLC).

### Data collection

The eligible studies were reviewed, and the data were extracted independently by two authors (ZQC and SLZ). The study information (the first author, the year of publication), study participants (the histological type of CRC, gender, mean age, and sample sizes), the characteristics of treatment (surgery, chemotherapy, radiotherapy), the characteristics of TGF-β (gene subtypes, test samples, test content, test methods), and prognostic outcomes of interest (OS and/or DFS) were extracted. If data from any of the above categories were not reported in the study, the item was recorded as “NR (not reported)”.

### Data analysis

Overall survival and disease-free survival were analysed separately for the eligible studies. The TGF-β value was classified as either “high expression” (overexpression) or “low expression”. For the quantitative aggregation of the survival times, the impact of TGF-β overexpression on survival times was measured. HRs and associated 95% CI were combined as effective values. If the HRs and 95% CI were given explicitly in the studies, we used the crude values. If these data were not given explicitly, they were calculated from the available numerical data or from the survival curve using the methods reported by Tierney. [19].

Heterogeneity of the individual HRs was calculated using Chi-square tests. A heterogeneity test with inconsistency index (*I* ^2^) statistic and Q statistic was performed. If the HRs were found to be homogenous, a fixed-effect model was used for analysis; if not, a random effect model was used. Subgroup analyses were performed for different countries (Asia, the West [Europe and America]) and analytical methods (univariate analysis, multivariate analysis). A *P* value ≤0.05 was considered statistically significant. An observed HR > 1 implied a worse prognosis in terms of high expression of TGF-β compared with low expression of TGF-β. The publication bias was evaluated using the methods of Begg [20]. All the calculations were performed using STATA version 12.0.

## Results

### Study characteristics

**Fig. 1:**
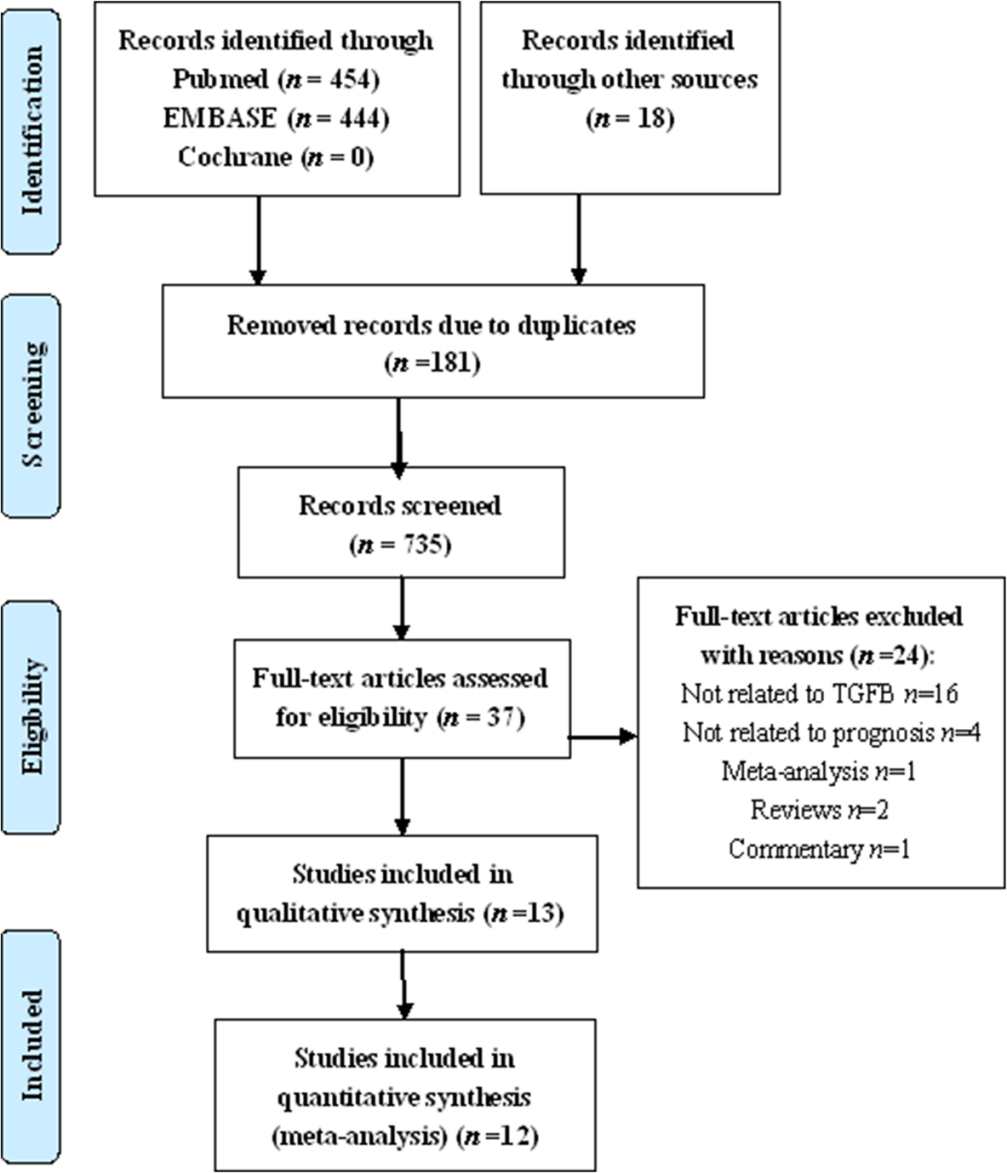
Flow chart of the search strategy

A total of 916 studies met the inclusion criteria (Fig. 1). In all, 181 studies were excluded as duplicates. The titles and abstracts of 735 studies were reviewed by two reviewers, and 698 studies that did not meet the inclusion criteria were excluded. The full texts of the remaining 37 studies were retrieved for review, and 24 studies were excluded after secondary screening. Eventually, 13 studies were included [10, 13, 15–17, 21–28]. The study conducted by Langenskiöld [23] was not included for analysis for the following reasons: (1) it had a great impact on the combined HR and accounted for 99% of the weight due to its small standard error of HR (0.005); (2) the study analysed colon cancer and rectal cancer separately; and (3) it is very difficult to get the small standard error in such a sample size (136). Therefore, 12 studies were eligible for this meta-analysis.

The major characteristics of the included studies are shown in Table 1 and Table 2. Nine studies were conducted in European countries (UK, Germany, Italy, Sweden, Poland, Greece and Bulgaria) and in the United States (US), and four were conducted in Asian countries (China, Japan and Korea) [21, 22, 25, 26]. All the eligible studies were published between 1995 and 2015. The sample size of the included studies ranged from 34 to 206 patients (median: 124 patients). In all, 1622 CRC patients were included. All patients included in the eligible studies underwent surgical resection. Only one study investigated rectal cancer [22], and two investigated colon cancer [25, 27], whereas other studies investigated colorectal cancer. One study included stage III patients [22], and one study included stage I-III patients [23]. Other studies included patients with all stages of CRC.

**Table 1:** The main characteristics of the included studies

Author	Year of publication	Country	Time	Sample size	Males	Mean of age (range)	Patients	Stage (UICC)	Differentiation	Surgery	Chemo−/Radio-therapy	Follow-up time (median, months)
Zhu J	2015	China	2003–2009	115	70	62 (29, 89)	CRC	All	All	All	NR/NR	NR
Chun HK	2014	Korea	2006–2007	201	132	58.6	Rectal cancer	III	All	All	Partial/Partial	57.8
Langenskiöld M	2013	Sweden	1999–2003	136	78	75 (44, 91)	CRC	I-III	All	All	Partial/No	51.0
Uhlmann ME	2012	Germany	1998–2001	103	67	66.7	CRC	All	NR	All	NR/NR	104.5
Lampropoulos P	2012	Greece	2005–2006	195	103	68.6 (36, 90)	CRC	All	NR	All	No/No	56.0
Li X	2011	China	2003–2004	147	86	NR (24, 87)	Colon cancer	All (Duke’s stage)	NR	All	Partial/No	≥ 60.0
Khanh do T	2011	Japan	1998–2005	206	114	63.3 (19, 92)	CRC	All	All	All	Partial/NR	≥ 60.0
Wincewicz A	2010	Poland	2004–2008	72	35	NR	CRC	All	Moderate and poor	All	NR/NR	NR
Gulubova M	2010	Bulgaria	1997–2006	142	92	65 ^b^ (39, 96)	CRC	All	All	All	NR/NR	37.6
Bellone G	2010	Italy	2004–2008	75	47	NR (24, 87)	Colon cancer^a^	All (Duke’s stage)	NR	All	NR/NR	44.0
Tsamandas AC	2004	Greece	1990–1998	124	79	66 ^b^ (25, 82)	CRC	All	All	All	Partial/Partial	68.0
Robson H	1996	UK	NR	72	47	70 (32, 92)	CRC	All	All	All	NR/NR	≥ 36.0
Friedman E	1995	US	1994	34	18	64.6	CRC	NR	NR	All	NR/NR	57.3 (mean)

*CRC* Colorectal cancer, *NR* not reported

^a^Only included adenoma and adenocarcinoma of the colon

^b^Median of age

**Table 2:** The TGF-β information and results of the included studies

	Gene subtype	High expression (%)	Test sample	Test content	Test method	Analytic method	Outcome	HR for OS	95% CI HR for OS	HR for DFS	95% CI HR for DFS
Zhu J	TGF-β1	53.0	Tissue	Protein	IHC	Multivariate	OS	3.33	1.41–7.87	NR	NR
Chun HK	TGF-β1	14.9	Tissue	Protein	IHC	Multivariate	DFS	NR	NR	9.19	1.26–67.20
Langenskiöld M	TGF-β1	NR	Tissue	Protein	ELISA	Multivariate	OS	1.00	0.99–1.01	NR	NR
Uhlmann ME	TGF-β	25.2	Tissue	mRNA	PCR	Surv curve	OS	1.13	0.42–3.03	NR	NR
Lampropoulos P	TGF-β	71.3	Tissue	Protein	IHC	Multivariate	OS	4.68	2.09–10.48	NR	NR
Li X	TGF-β1	70.7	Tissue	Protein	IHC	Univariate	OS, DFS	0.75	0.48–1.16	0.81	0.55–1.20
Khanh do T	TGF-β1	55.3	Tissue	Protein	IHC	Multivariate	OS, RFS	1.62	0.74–3.56	1.46	0.74–2.87
Wincewicz A	TGF-β1	83.3	Tissue	Protein	IHC	Survival curve	DFS	NR	NR	1.00	0.93–1.07
Gulubova M	TGF-β1	25.4	Tissue	Protein	IHC	Surv curve	OS	1.35	0.82–2.23	NR	NR
Bellone G	TGF-β1	NR	Serum	Protein	ELISA	Multivariate	DFS	NR	NR	1.11	1.03–1.20
Tsamandas AC	TGF-β1	79.0	Tissue	Protein	IHC	Multivariate	OS, DFS	1.55	1.33–3.08	1.09^a^	0.27–4.4
Robson H	TGF-β1	58.3	Tissue	Protein	IHC	Surv curve	OS	0.56	0.15–2.14	NR	NR
Friedman E	TGF-β1	58.8	Tissue	Protein	PCR	Surv curve	DFS	NR	NR	3.33	0.47–23.43

*DFS* disease-free survival, *ELISA* enzyme-linked immunosorbent assay, *IHC* Immunohistochemistry, *Multivariate* Multivariate survival analyses, *NR* not reported, *OS* overall survival, *PCR* Polymerase chain reaction, *RFS* recurrence-free survival (which was used as DFS), *Surv curve* survival curve, *Univariate* Univariate survival analyses

^a^:the result was analysed by the Univariate method

Eight studies reported the prognostic value of TGF-β with respect to OS in CRC patients (Table 2). Of the 8 studies, 6 directly reported the HRs, while the other 3 studies provided survival curves. Three studies identified high expression of TGF-β as an indicator of poor prognosis in terms of OS [13, 16, 21], whereas others showed no significant difference. Seven studies reported the prognostic value of TGF-β for DFS in CRC patients (Table 2). Of these 7 studies, 5 directly reported the HRs, while the other 2 studies provided survival curves. Two out of the 7 studies identified high expression of TGF-β as an indicator of poor prognosis in terms of DFS [22, 27], whereas others showed no significant difference.

### Meta-analysis of OS

**Fig. 2:**
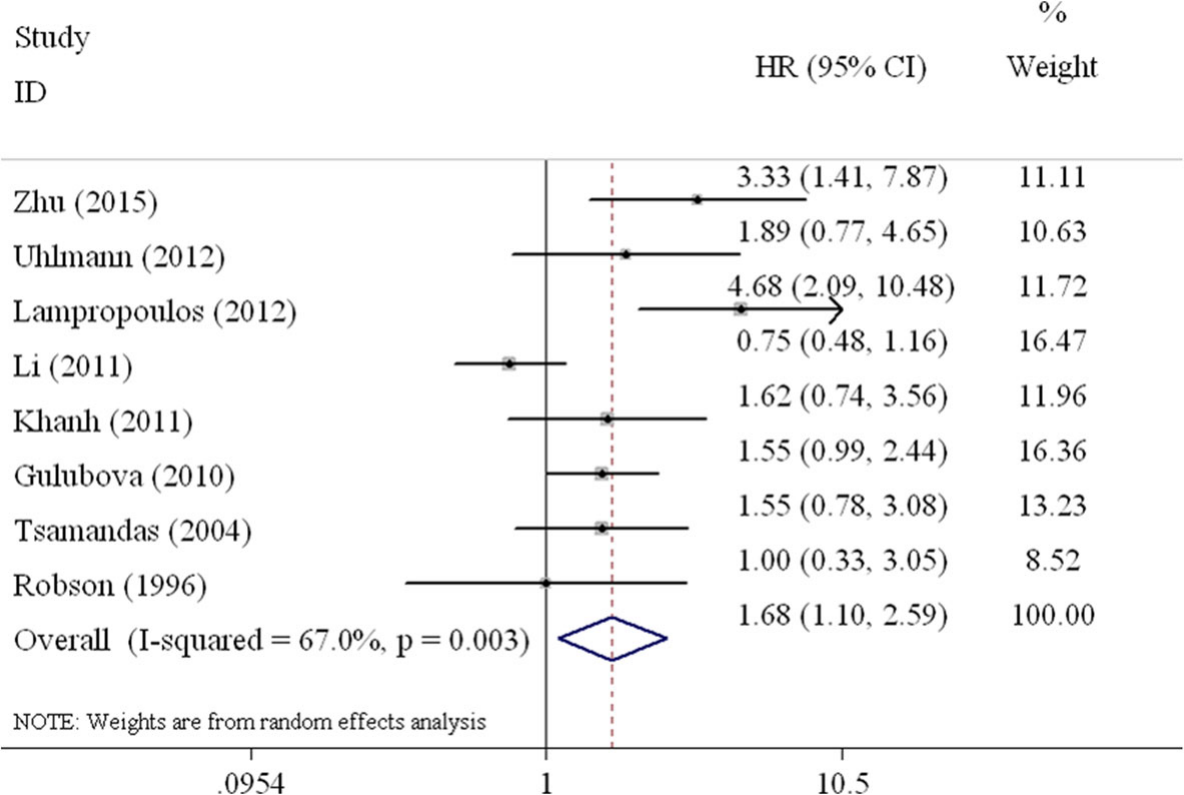
Forest plot evaluating the combined HR between TGF-β and OS for all included studies

**Fig. 3:**
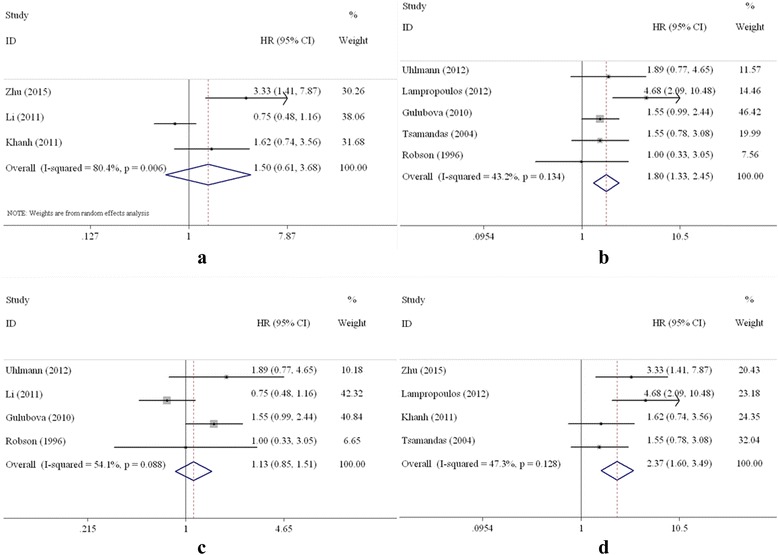
Forest plot of subgroup analysis on TGF-β and OS. **a** Asian countries; **b** Western countries; **c** univariate analysis; **d** multivariate analysis

Eight studies that focused on the relationship of TGF-β expression to overall survival of CRC patients undergoing surgery were included in the meta-analysis [10, 13, 15, 16, 21, 24–26]. The combined HR value of the 8 studies that evaluated the high expression of TGF-β with respect to OS was 1.68 (95% CI: 1.10–2.59, Table 3, Fig. 2), which indicates that high expression of TGF-β was associated with a poor OS of patients with CRC. When the subgroups were analysed based on country, the combined HRs of the Asian studies and the Western studies were 1.50 (95% CI: 0.61–3.68) and 1.80 (95% CI: 1.33–2.45), respectively (Fig. 3). Subgroup analyses were performed according to the analytical method of the individual studies. The combined HR of the studies based on multivariate analysis was 2.37 (95% CI: 1.60–3.49; Fig. 3). However, the relationship between TGF-β overexpression and OS was not statistically significant (HR = 1.13, 95% CI: 0.85–1.51; Fig. 3) according to the univariate analysis.

**Table 3:** Results of the meta-analysis

	Number of studies	Number of patients	HR (95% CI)	Heterogeneity (I^2^, χ^2^, P)
Overall survival				
All	8	1119	1.68 (1.10–2.59)^a^	67.0%, 21.21, 0.003
Univariate analysis	4	479	1.13 (0.85–1.51)	54.1%, 6.54, 0.088
Multivariate analysis	4	640	2.37 (1.60–3.49)	47.3%, 5.69, 0.128
Country				
Asian	3	468	1.50 (0.61–3.68)^a^	80.4%, 10.21, 0.006
Western	5	651	1.80 (1.33–2.45)	43.2%, 7.04, 0.134
Disease-free survival				
All	7	859	1.11 (1.03–1.19)	33.0%, 8.96, 0.176
Univariate analysis	4	377	0.86 (0.60–1.24)	0.0%, 2.18, 0.536
Multivariate analysis	3	482	1.12 (1.04–1.21)	59.4%, 4.92, 0.085
Country				
Asian	3	554	1.42 (0.61–3.31)^a^	71.6%, 7.04, 0.030
Western	4	305	1.11 (1.03–1.20)	0.0%, 1.56, 0.667

^a^The result was based on the random effect model

### Meta-analysis of DFS

**Fig. 4:**
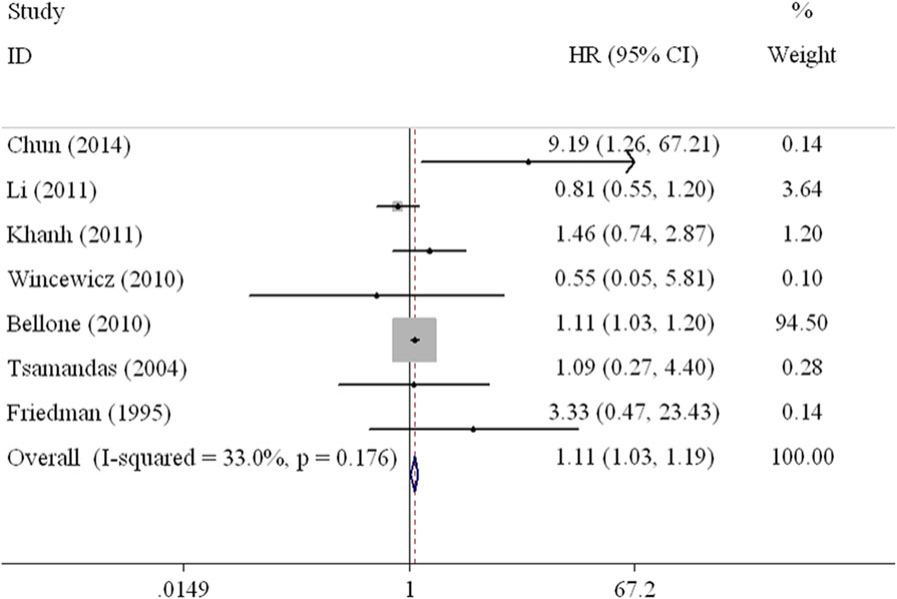
Forest plot evaluating the combined HR between TGF-β and DFS for all included studies

Seven studies on TGF-β and DFS in CRC patients undergoing surgery were included in the meta-analysis [16, 17, 22, 25–28]. The combined HR of the 7 studies that evaluated the relationship of the high expression of TGF-β to DFS was 1.11 (95% CI: 1.03–1.19, Table 3, Fig. 4), which suggests that high expression of TGF-β is a significant prognostic factor for CRC patients.

**Fig. 5:**
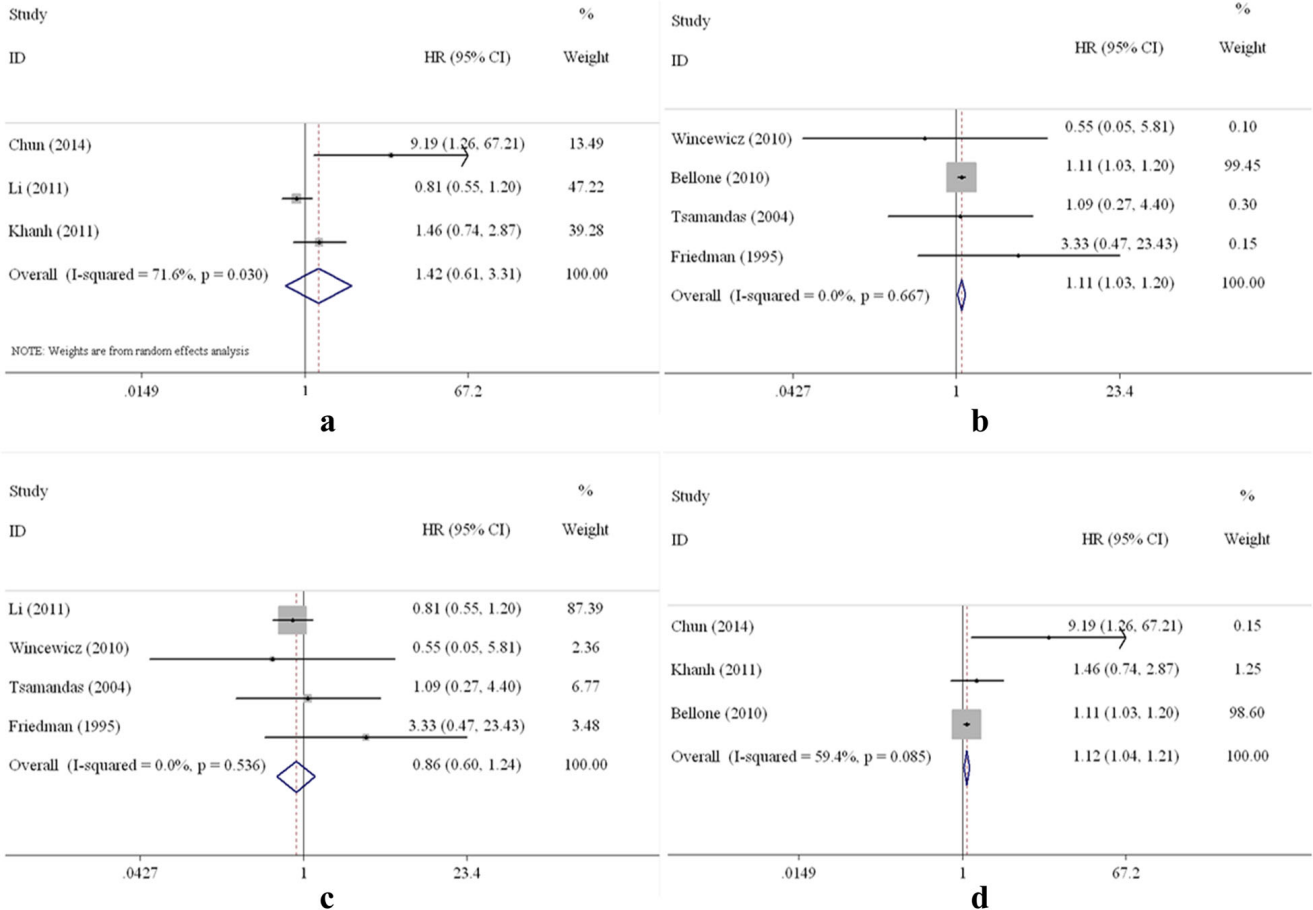
Forest plot of subgroup analysis on TGF-β and DFS. **a** Asian countries; **b** Western countries; **c** univariate analysis; **d** multivariate analysis)

When the subgroups were analysed based on country, the combined HRs of the Asian and Western studies were 1.42 (95% CI: 0.61–3.31) and 1.11 (95% CI: 1.03–1.20), respectively (Fig. 5). The combined HR of the studies based on multivariate analysis was 1.12 (95% CI: 1.04–1.21; Fig. 5). However, statistical significance was not observed with respect to the association of TGF-β overexpression and DFS (HR = 0.86, 95% CI: 0.60–1.24; Fig. 5) according to the univariate analysis.

**Fig. 6:**
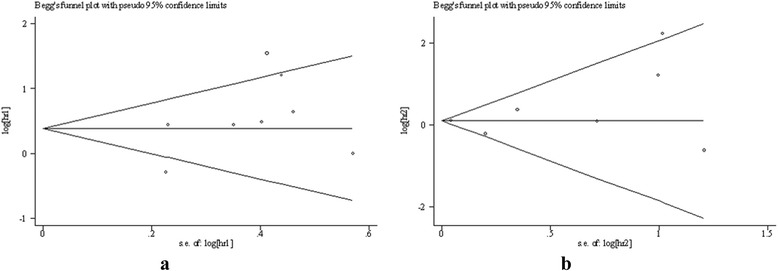
Funnel plot for the included studies. **a** OS; **b** DFS

The Begg’s funnel plot and Egger’s test were performed to evaluate the publication bias of the included studies (Fig. 6). Eight studies that investigated the effect of high expression of TGF-β on OS yielded a slope of −0.51 (95% CI: -1.96–0.94) with no significant difference (*P* = 0.423). Seven studies that investigated the effect of high expression of TGF-β on DFS yielded a slope of 0.08 (95% CI: -0.07–0.23) with no significant difference (*P* = 0.231). These results indicate the absence of publication bias in the included studies.

## Discussion

High expression of TGF-β in primary CRC is associated with advanced stages of the disease, a greater likelihood of recurrence and decreased survival [15, 28]. TGF-β stimulates proliferation and invasion in advanced stages of CRC and leads to distant metastasis [29]. Many studies have been conducted to assess the prognostic value of TGF-β in patients with CRC, but the conclusions have been inconclusive.

Our analysis showed that high expression of TGF-β was a prognostic indicator in CRC patients undergoing surgery. With respect to OS, the mortality rate of patients with a high expression of TGF-β was 1.68 times that of patients with a low expression. With respect to DFS, the mortality rate of patients with a high expression of TGF-β was 1.11 times that of patients with a low expression. Our results were consistent with those of studies of other cancers. The results of the meta-analysis conducted by Yang demonstrated that the high expression of TGF-β was strongly associated with the 3-year survival rate in patients with glioma [30]. Similar results were also found in patients with gastric cancer [31], hepatocellular carcinoma [32], renal cancer [33], breast cancer [34], and oesophageal cancer [35].

Determination of TGF-β expression independently provided valuable prognostic information in relation to two targeting pathways in patients with CRC. (1) One piece of information was related to the following signalling pathway molecules: matrix metalloproteinase-2 (MMP-2), cyclooxygenase-2 (COX-2), vascular endothelial growth factor (VEGF) and TGF-β. TGF-β acted as a tumour promoter in advanced stages of CRC, which potentially led to increased expression of MMP-2 and COX-2 [36, 37]. Coordinated, increased expression of COX-2, TGF-β and VEGF have been associated with increased angiogenesis, which in turn has been described to be a prerequisite for tumour growth [24, 38]. The meta-analysis showed that high expression levels of MMP-2, COX-2 and VEGF were associated with decreased survival time for CRC patients [39–41]. (2) Another piece of information involved the Smad4 and VEGF-C signalling pathway. Upon stimulation by TGF-β1, Smad2/Smad3 is phosphorylated by activated TGF-β receptors and forms a complex with Smad4. Smad4 translocates into the nucleus, where it affects transcription of the VEGF-C gene [25]. The meta-analysis demonstrated that high expression of VEGF-C was associated with decreased OS of patients with CRC [42].

TGF-β had a stronger association with OS and DFS in CRC patients undergoing surgery in Western countries than in Asian countries. The results suggested that CRC patients in Western countries who have overexpression of TGF-β are at a higher risk of death than those in Asian countries. With respect to DFS, the HRs among Asian countries were significantly different, whereas those among Western countries were not. This discrepancy may be related to heterogeneity and different disease characteristics in the Asian studies. (1) Heterogeneity was present among the Asian studies for DFS, and the *P* values were less than 0.05. For example, the HR of the study by Chun was 9.19 [22], but the HR for the study by Li was 0.81 [25]. (2) Different disease characteristics were also observed in the Asian studies. Two Asian studies involved CRC [21, 26], one involved rectal cancer [22], and one involved colon cancer [25]. One study enrolled CRC patients with stage III disease (AJCC) only [22]. Three other studies enrolled CRC patients with all stages of the disease [21, 25, 26]. When using disease (colorectal, rectal, and colon) as a grouping factor, the effects of TGF-β on prognosis were inconsistent. The HR of TGF-β in colorectal cancer was much higher than that of colon cancer.

Subgroup analysis was also performed based on the analytical method (univariate analysis, multivariate analysis). The HR of TGF-β in the multivariate analysis (2.37 for OS, 1.12 for DFS) was higher than that in the univariate analysis (1.13 for OS, 0.86 for DFS). If the variable was not significantly different in the univariate analysis, the variable was not entered into the Cox proportional hazards model (multivariate analysis). To understand the independent effect of TGF-β expression on prognosis of patients with CRC, multivariate analysis should be used to control the effects of other possible risk factors (e.g., gender, tumour grade, TNM staging system). The effect of the high expression of TGF-β on prognosis based on the univariate analysis was confounded because prognosis was affected by other factors.

Several limitations should be considered. (1) The method of therapy greatly affected the survival time of CRC patients. Although the use of chemotherapy or radiotherapy differed substantially among the included studies, all the included CRC patients were treated with surgery. Thus, the confounding effects of different therapeutic modalities would not be substantial. (2) The second limitation was the heterogeneity of the eligible studies. The results of subgroup analyses suggested that heterogeneity may have been partly due to the following variables: diversity of the disease and the countries where the studies were conducted. Other variables, such as follow-up time and the non-standardised methodologies for the assessment of TGF-β, among others, may be related to the heterogeneity. However, these subgroup analyses were not conducted. (3) The study conducted by Langenskiöld was excluded due to its great impact on the combined HR [23]. If the study was included in the analysis, it would have accounted for 99% of the weight due to its small standard error for HR (0.005). (4) TGF-β1 was not assessed in all the included studies. Two studies reported that they included the TGF-β gene, but it was unclear whether the gene was TGF-β1 [13, 24].

## Conclusions

This meta-analysis provides evidence that high expression of TGF-β is significantly associated with worse OS and DFS in CRC patients who undergo surgery. TGF-β could be used as a prognostic biomarker in colorectal cancer. Subgroup analysis indicates that high expression of TGF-β is associated with cancer progression in CRC patients from Western countries. However, high expression of TGF-β was not associated with cancer progression in Asian patients with CRC due to the high heterogeneity of the included studies. These results can guide postoperative treatment of CRC patients, especially the application of chemotherapy in CRC patients from Western countries.

## Additional files

Additional file 1: PRISMA 2009 Checklist. (DOC 66 kb)

Additional file 2: The search strategy. (DOC 27 kb)

## Abbreviations

CI: confidence interval
COX-2: cyclooxygenase-2
CRC: colorectal cancer
DFS: disease-free survival
ELISA: enzyme-linked immunosorbent assay
HRs: hazard ratios
IHC: immunohistochemistry
MMP-2: matrix metalloproteinase-2
OS: overall survival
PCR: polymerase chain reaction
RFS: relapse-free survival
TGF-β: Transforming growth factor-beta
VEGF: vascular endothelial growth factor

## References

[1] Siegel R, Naishadham D, Jemal A (2013). Cancer statistics, 2013. CA Cancer J Clin.

[2] Chen W, Zheng R, Baade PD, Zhang S, Zeng H, Bray F, Jemal A, Yu XQ, He J (2016). Cancer statistics in China, 2015. CA Cancer J Clin.

[3] Brenner H, Kloor M, Pox CP (2014). Colorectal cancer. Lancet.

[4] Ferlay J, Shin HR, Bray F, Forman D, Mathers C, Parkin DM (2010). Estimates of worldwide burden of cancer in 2008: GLOBOCAN 2008. Int J Cancer.

[5] Erstad DJ, Tumusiime G, Cusack JC (2015). Prognostic and predictive biomarkers in colorectal cancer: implications for the clinical surgeon. Ann Surg Oncol.

[6] Ku GY, Haaland BA, de Lima LG, Jr. (2012). Cetuximab in the first-line treatment of K-ras wild-type metastatic colorectal cancer: the choice and schedule of fluoropyrimidine matters. Cancer Chemother Pharmacol.

[7] Marien KM, Croons V, Martinet W, De Loof H, Ung C, Waelput W, Scherer SJ, Kockx MM, De Meyer GR (2015). Predictive tissue biomarkers for bevacizumab-containing therapy in metastatic colorectal cancer: an update. Expert Rev Mol Diagn.

[8] Sanz-Garcia E, Grasselli J, Argiles G, Elez ME, Tabernero J (2016). Current and advancing treatments for metastatic colorectal cancer. Expert Opin Biol Ther.

[9] Thapa N, Lee BH, Kim IS (2007). TGFBIp/betaig-h3 protein: a versatile matrix molecule induced by TGF-beta. Int J Biochem Cell Biol.

[10] Gulubova M, Manolova I, Ananiev J, Julianov A, Yovchev Y, Peeva K (2010). Role of TGF-beta1, its receptor TGFbetaRII, and Smad proteins in the progression of colorectal cancer. Int J Color Dis.

[11] Lin RL, Zhao LJ (2015). Mechanistic basis and clinical relevance of the role of transforming growth factor-beta in cancer. Cancer Biol Med.

[12] Bachman KE, Park BH (2005). Duel nature of TGF-beta signaling: tumor suppressor vs. tumor promoter. Curr Opin Oncol.

[13] Lampropoulos P, Zizi-Sermpetzoglou A, Rizos S, Kostakis A, Nikiteas N, Papavassiliou AG (2012). Prognostic significance of transforming growth factor beta (TGF-beta) signaling axis molecules and E-cadherin in colorectal cancer. Tumour Biol.

[14] Yashiro M, Hirakawa K, Boland CR (2010). Mutations in TGFbeta-RII and BAX mediate tumor progression in the later stages of colorectal cancer with microsatellite instability. BMC Cancer.

[15] Robson H, Anderson E, James RD, Schofield PF (1996). Transforming growth factor beta 1 expression in human colorectal tumours: an independent prognostic marker in a subgroup of poor prognosis patients. Br J Cancer.

[16] Tsamandas AC, Kardamakis D, Ravazoula P, Zolota V, Salakou S, Tepetes K, Kalogeropoulou C, Tsota I, Kourelis T, Makatsoris T (2004). The potential role of TGFbeta1, TGFbeta2 and TGFbeta3 protein expression in colorectal carcinomas. Correlation with classic histopathologic factors and patient survival. Strahlenther Onkol.

[17] Wincewicz A, Koda M, Sulkowski S, Kanczuga-Koda L, Sulkowska M (2010). Comparison of beta-catenin with TGF-beta1, HIF-1alpha and patients' disease-free survival in human colorectal cancer. Pathol Oncol Res.

[18] Moher D, Liberati A, Tetzlaff J, Altman DG (2009). Preferred reporting items for systematic reviews and meta-analyses: the PRISMA statement. J Clin Epidemiol.

[19] Tierney JF, Stewart LA, Ghersi D, Burdett S, Sydes MR (2007). Practical methods for incorporating summary time-to-event data into meta-analysis. Trials.

[20] Begg CB, Mazumdar M (1994). Operating characteristics of a rank correlation test for publication bias. Biometrics.

[21] Zhu J, Chen X, Liao Z, He C, Hu X (2015). TGFBI protein high expression predicts poor prognosis in colorectal cancer patients. Int J Clin Exp Pathol.

[22] Chun HK, Jung KU, Choi YL, Hong HK, Kim SH, Yun SH, Kim HC, Lee WY, Cho YB (2014). Low expression of transforming growth factor beta-1 in cancer tissue predicts a poor prognosis for patients with stage III rectal cancers. Oncology.

[23] Langenskiöld M, Ivarsson ML, Holmdahl L, Falk P, Kåbjörn-Gustafsson C, Angenete E (2013). Intestinal mucosal MMP-1 -a prognostic factor in colon cancer. Scand J Gastroenterol.

[24] Uhlmann ME, Georgieva M, Sill M, Linnemann U, Berger MR (2012). Prognostic value of tumor progression-related gene expression in colorectal cancer patients. J Cancer Res Clin Oncol.

[25] Li X, Liu B, Xiao J, Yuan Y, Ma J, Zhang Y (2011). Roles of VEGF-C and Smad4 in the lymphangiogenesis, lymphatic metastasis, and prognosis in colon cancer. J Gastrointest Surg.

[26] Khanh do T, Mekata E, Mukaisho K, Sugihara H, Shimizu T, Shiomi H, Murata S, Naka S, Yamamoto H, Endo Y (2011). Prognostic role of CD10+ myeloid cells in association with tumor budding at the invasion front of colorectal cancer. Cancer Sci.

[27] Bellone G, Gramigni C, Vizio B, Mauri FA, Prati A, Solerio D, Dughera L, Ruffini E, Gasparri G, Camandona M (2010). Abnormal expression of Endoglin and its receptor complex (TGF-beta1 and TGF-beta receptor II) as early angiogenic switch indicator in premalignant lesions of the colon mucosa. Int J Oncol.

[28] Friedman E, Gold LI, Klimstra D, Zeng ZS, Winawer S, Cohen A (1995). High levels of transforming growth factor beta 1 correlate with disease progression in human colon cancer. Cancer Epidemiol Biomark Prev.

[29] Xu Y, Pasche B: TGF-beta signaling alterations and susceptibility to colorectal cancer. Hum Mol Genet 2007, 16 Spec No 1 R14-R20. 10.1093/hmg/ddl486 PMC2637552 17613544

[30] Yang X, Lv S, Zhou X, Liu Y, Li D, Shi R, Kang H, Zhang J, Xu Z (2014). The clinical implications of transforming growth factor Beta in pathological grade and prognosis of Glioma patients: a meta-analysis. Mol Neurobiol.

[31] Tas F, Yasasever CT, Karabulut S, Tastekin D, Duranyildiz D (2015). Serum transforming growth factor-beta1 levels may have predictive and prognostic roles in patients with gastric cancer. Tumour Biol.

[32] Wang Y, Liu T, Tang W, Deng B, Chen Y, Zhu J, Shen X (2016). Hepatocellular carcinoma cells induce regulatory T cells and lead to poor prognosis via production of transforming growth factor-beta1. Cell Physiol Biochem.

[33] Lebdai S, Verhoest G, Parikh H, Jacquet SF, Bensalah K, Chautard D, Rioux Leclercq N, Azzouzi AR, Bigot P (2015). Identification and validation of TGFBI as a promising prognosis marker of clear cell renal cell carcinoma. Urol Oncol.

[34] Bahhnassy A, Mohanad M, Shaarawy S, Ismail MF, El-Bastawisy A, Ashmawy AM, Zekri AR (2015). Transforming growth factor-beta, insulin-like growth factor I/insulin-like growth factor I receptor and vascular endothelial growth factor-a: prognostic and predictive markers in triple-negative and non-triple-negative breast cancer. Mol Med Rep.

[35] Ozawa D, Yokobori T, Sohda M, Sakai M, Hara K, Honjo H, Kato H, Miyazaki T, Kuwano H (2016). TGFBI expression in cancer Stromal cells is associated with poor prognosis and Hematogenous recurrence in esophageal Squamous cell carcinoma. Ann Surg Oncol.

[36] Massague J (2008). TGFbeta in cancer. Cell.

[37] Neil JR, Johnson KM, Nemenoff RA, Schiemann WP (2008). Cox-2 inactivates Smad signaling and enhances EMT stimulated by TGF-beta through a PGE2-dependent mechanisms. Carcinogenesis.

[38] Fosslien E (2001). Review: molecular pathology of cyclooxygenase-2 in cancer-induced angiogenesis. Ann Clin Lab Sci.

[39] Shi M, Yu B, Gao H, Mu J, Ji C (2013). Matrix metalloproteinase 2 overexpression and prognosis in colorectal cancer: a meta-analysis. Mol Biol Rep.

[40] Peng L, Zhou Y, Wang Y, Mou H, Zhao Q: Prognostic significance of COX-2 immunohistochemical expression in colorectal cancer: a meta-analysis of the literature. *PLoS One* 2013, 8(3):e58891. 10.1371/journal.pone.0058891 PMC3604072 23527044

[41] Des Guetz G, Uzzan B, Nicolas P, Cucherat M, Morere JF, Benamouzig R, Breau JL, Perret GY (2006). Microvessel density and VEGF expression are prognostic factors in colorectal cancer. Meta-analysis of the literature. Br J Cancer.

[42] Zong S, Li H, Shi Q, Liu S, Li W, Hou F (2016). Prognostic significance of VEGF-C immunohistochemical expression in colorectal cancer: a meta-analysis. Clin Chim Acta.

