# Supplementary material for: Prognostic value of transforming growth factor-beta in patients with colorectal cancer who undergo surgery: a meta-analysis
Source: BMC Cancer. 2017 Apr 4;17:240. doi: 10.1186/s12885-017-3215-7 (PMC5379512; doi:10.1186/s12885-017-3215-7)
Supplement: Supplementary file 2 — The search strategy. (DOC 27 kb) [file 12885_2017_3215_MOESM2_ESM.doc]

**Additional file 2. The search strategy**

All the searches were performed on March 8th 2016.

**Pubmed**

1. “colorectal” OR “large intestine” OR “large bowel” OR “colon” OR “colonic” OR “rectal” OR “rectum” [MeSH Terms] (389 304 articles)

2. “colorectal” OR “large intestine” OR “large bowel” OR “colon” OR “colonic” OR “rectal” OR “rectum” [Title/Abstract] (388 334 articles)

3. #1 OR #2 (394 691 articles)

4. “cancer” OR “carcinoma” OR “tumor” OR “tumour” OR “neoplasm” OR “cancers” [MeSH Terms] (2 757 849 articles)

5. “cancer” OR “carcinoma” OR “tumor” OR “tumour” OR “neoplasm” OR “cancers” [Title/Abstract] (2 776 126 articles)

6. #4 OR #5 (2 776 126 articles)

7. #3 AND #6 (210 278 articles)

8. “TGFβ” OR “TGFβ1” OR “Transforming growth factor" [Title/Abstract] (56,165 articles)

9. “prognosis” OR “prognoses” OR “prognostic” OR “predictive” OR “biomarker” OR “marker” OR “survival” OR “survive” OR “Cox” OR “Log-rank” OR “Kaplan-Meier” [Title/Abstract] (3 480 569 articles)

10. #7 AND #8 AND #9 (454 articles)

**Embase**

1. "colorectal Neoplasms"/exp OR "[colonic Neoplasms](http://www.ncbi.nlm.nih.gov/mesh?Db=mesh&Term="Colonic Neoplasms "%5BMESH%5D)"/exp OR "[Rectal Neoplasms](http://www.ncbi.nlm.nih.gov/mesh?Db=mesh&Term="Rectal Neoplasms "%5BMESH%5D)"/exp (272,720 articles)

2. TGFB1 OR "Transforming growth factor" (111,978 articles)

3. prognosis OR prognoses OR prognostic OR predictive OR biomarker OR marker OR survival OR survive OR Cox OR Logrank OR Kaplan-Meier (2,665,001 articles)

4. #1 AND #2 AND #3 (444 articles)

**Cochrane**

1. "colorectal Neoplasms" OR "[colonic Neoplasms](http://www.ncbi.nlm.nih.gov/mesh?Db=mesh&Term="Colonic Neoplasms "%5BMESH%5D)" OR "[Rectal Neoplasms](http://www.ncbi.nlm.nih.gov/mesh?Db=mesh&Term="Rectal Neoplasms "%5BMESH%5D)" (6,052 articles)

2. TGFB1 OR "Transforming growth factor" (2 articles)

3. prognosis OR prognoses OR prognostic OR predictive OR biomarker OR marker OR survival OR survive OR Cox OR Logrank OR Kaplan-Meier (103,210 articles)

4. #1 AND #2 AND #3 (0 articles)
